# Peppermint essential oil inhibits *Drosophila suzukii* emergence but reduces *Pachycrepoideus vindemmiae* parasitism rates

**DOI:** 10.1038/s41598-020-65189-5

**Published:** 2020-06-04

**Authors:** Chelsea Megan Gowton, Michał Reut, Juli Carrillo

**Affiliations:** 1 Faculty of Land and Food Systems, Centre for Sustainable Food Systems, Biodiversity Research Centre, The University of British Columbia, Unceded xʷməθkʷəy̓əm (Musqueam) Territory, Vancouver, V6T 1Z4 British Columbia Canada; 2 Department of Applied Entomology, Faculty of Horticulture and Landscape Architecture, Warsaw University of Life Sciences – SGGW, Nowoursynowska 159, 02-776 Warsaw, Poland

**Keywords:** Agroecology, Entomology

## Abstract

Spotted Wing Drosophila (*Drosophila suzukii*; Matsumura) is an invasive fruit fly with the ability to oviposit in a broad range of agriculturally valuable fruits. Volatile organic compounds (VOCs) produced by botanical oils may reduce *D. suzukii*’s attraction to hosts and decrease survival, but it is unknown whether their efficacy varies across *D. suzukii* life stages or affects the survival and success of higher trophic levels. Through a series of laboratory bioassays, we evaluated the effects of peppermint (*Mentha arvensis* L.) oil produced VOCs on *D. suzukii* survival and the survival of and parasitism rates by a pupal parasitoid wasp, *Pachycrepoideus vindemmiae* (Rondani). First, we determined whether fumigation with peppermint oil VOCs at the pupal stage reduced adult emergence, and whether this depended on environmental conditions (i.e. soil moisture). Second, we evaluated whether fumigation with peppermint oil VOCs reduced or enhanced parasitism by the pupal parasitoid and whether this depended on the timing of peppermint oil VOC exposure (i.e. before, during, or after parasitoid access). Fumigation with VOCs of 4.5 mg of peppermint oil reduced *D. suzukii* emergence under moist soil conditions but dry soil had a similar effect on reducing adult emergence as peppermint oil presence. Peppermint oil VOC fumigation was toxic to adult *P. vindemmiae*, but developing *P. vindemmiae* were unaffected by peppermint oil VOC fumigation. Using peppermint essential oil as a fumigant may reduce *D. suzukii* emergence from the pupal stage. However, this could negatively impact *P. vindemmiae* dependent on the timing of application.

## Introduction

Spotted Wing Drosophila (*Drosophila suzukii*; Matsumura) is a fruit fly native to East Asia and originally described from infested cherries in Japan^1^. The female has a serrated ovipositor not commonly found in other drosopholid flies^2^, which allows it to penetrate into a broad range of agriculturally valuable fruits, including cherry, blueberry, blackberry, strawberry, raspberry, apricot and grapes^3,4^. Before *D. suzukii* management regimes were established, west coast US growers experienced substantial yield losses (up to 50% in raspberries) due to *D. suzukii* infestation^5^. Potential economic impact in the absence of management action is estimated to be as high as 421.5 million USD annually^5^ with other estimates suggesting even greater revenue loss due to inflation and other market factors^6^. In order to reduce *D. suzukii* populations, growers typically rely on pesticide sprays consisting of oganophosphates, carbamates, pyrethroids, and spinosad which are mostly effective at targeting gravid females^3^. Fruit processors reject infested fruit, thus, current management practices in the Pacific Northwest rely on synthetic insecticide sprays every four to seven days^7^. There has been increasing concern for pesticide resistance evolution, after recent studies have shown decreased susceptibility to spinosad^8^ and inherited spinosad tolerance in a Watsonville, California, *D. suzukii* population^9^. Other management options include cultural controls, such as netting and hand picking of dropped fruit; these can be both expensive and labour-intensive. There is a strong need for additional research into alternative methods of control across multiple life stages of *D. suzukii*.

Volatile organic compounds (VOCs) that mimic host plants and ripening fruit can attract *D. suzukii*^10–14^, and several VOCs can deter adult *D. suzukii*^15–20^. In the field, dispensers of 1-octen-3-ol, a volatile molecule produced by fungi and some plants, reduced *D. suzukii* oviposition by 41.5% and the number of adults in fruit clusters near the dispenser by 47.6%^15^. Botanical oils which contain high concentrations of VOCs, such as peppermint (*Mentha × piperita*, Lamiaceae) oil, can repel adults or, in the case of thyme oil, increase adult mortality^19^. Extending this, Renkema *et al*.^20^ found reduced larval infestations in strawberries that were surrounded by menthol-infused polymer flakes. While peppermint essential oil has been reported to repel adult *D. suzukii*^19,20^, it has yet to be determined if these botanical extracts are toxic to other *D. suzukii* life stages.

As adults only comprise 8% of the population at any given time^21^, management of *D. suzukii* could be improved through targeting larvae and pupae. This can include maintaining bare ground or using black plastic around host plants, which can create a hostile microclimate for all life stages for *D. suzukii* (e.g. high surface temperatures^22^ and low humidity^23,24^), or using pesticides that target other life stages^25^. The use of natural enemies for biological control, including generalist predators and parasitoids, is another strategy for *D. suzukii* control^26^. Pupae may be especially vulnerable to natural enemies as 82–93% of *D. suzukii* pupae develop in soil and not in the fruit^27^, similar to the behaviour of other fruit-targeting *Drosophilids*^28^. In the field, predatory hemipterans have been found to occur in *D. suzukii*-infested fruit^29,30^ and Woltz and Lee^27^ found exposure to predators in a blueberry field to decrease the abundance of pupae by 61–91% and larval survival by 19–49%, due to multiple predators, including ants and spiders. Similarly, in a laboratory setting, Garbarra *et al*.^29^ found high levels of *D. suzukii* larval and pupal predation by the earwig (*Labidura riparia* Pallas).

Parasitic wasps are another biological control option for *D. suzukii* management, including the generalist pupal parasitoid *Pachycrepoideus vindemmiae* (Rondani; family: Pteromalidae). *P. vindemmiae* is able to parasitize over 60 fly species, including *D. suzukii*^29,31^ and several other *Drosophila* species^32^*. P. vindemmiae* has a broad native range across North America and Europe^29,33,34^, and natural populations of *P. vindemmiae* often overlap with *D. suzukii*-infested areas^35–37^. In the laboratory, individual *P. vindemmiae* can successfully lay 7.5 ± 0.64 eggs in *D. suzukii* pupae each day^29^, yet current in-field parasitism rates are reported to be less than 10% in wild *D. suzukii* populations^38^. Furthermore *P. vindemmiae* experience high mortality and had lower parasitism rates when exposed to insecticides, including spinosyns, abamectin, neonicotinoids, organophosphates and pyrethroids^39^. However, it may be that optimising the use of resident parasitoids, such as *P. vindemmiae*, could reduce *D. suzukii* as part of an integrated pest management program.

Integrated pest management programs use multiple methods to reduce pest populations, including chemical and biological control^40^. Ideally, the multiple strategies used for pest control should work in synergy; however classical pesticide regimes can reduce the efficacy of biological control. Similarly, botanical oils and botanical oil VOCs may have both direct effects on natural enemy survival and performance, and also indirect effects through changes to host *D. suzukii* quality and susceptibility. We evaluated whether peppermint oil produced VOCs reduce *D. suzukii* pupal survival and whether this depended on soil moisture. We additionally assessed the compatibility of fumigation with peppermint oil VOCs with biological control, by evaluating the effects of peppermint oil produced VOCs on parasitoid wasp performance (survival, oviposition success, and adult emergence).

## Materials and methods

### Insects

*Drosophila suzukii* were kept in an incubation chamber set to 16:8 light:dark cycle with temperatures at 24.5 °C during light hours and 22.5 °C during dark hours. We reared *D. suzukii* on commercially available *Drosophila* diet (Ward’s Instant Drosophila medium) supplemented with instant yeast (Red Star). Our laboratory *D. suzukii* colony was founded in June 2017, by individuals sourced from another colony started in the summer 2016 with *D. suzukii* collected from blueberries in Agassiz, BC, Canada. The laboratory *D. suzukii* colony is supplemented annually with wild-caught individuals.

The laboratory *P. vindemmiae* colony began in May 2018, from a colony established in 2015 from collections in sweet cherry at Summerland, BC. *Pachycrepoideus vindemmiae* were kept in separate rearing containers in the same incubator as our *D. suzukii* colony. We fed our *P. vindemmiae* colony 10% sugar water solution and a protein-lipid supplement (high protein patty, MegaBee). *Drosophila suzukii* pupae were provided for *P. vindemmiae* colony oviposition three times a week.

For all experiments involving these insects, we used 2–3-day-old *D. suzukii* pupae and 3–5-day-old *P. vindemmiae* adults. To remove excess diet, we washed all *D. suzukii* pupae under tap water and allowed them to air dry for one hour before any experiments. Both *D. suzukii* and *P. vindemmiae* used in the experiments were naïve to peppermint oil produced VOCs, and the *P. vindemmiae* had not previously oviposited.

### Essential oil GC-MS analysis

Volatiles were collected from the headspace of a commercially available Japanese peppermint essential oil (*Mentha arvensis* L., Bulk Apothecary), with no dilution, and analyzed by headspace GC/MS at the Wine Research Centre Mass Spectrometry Core Facility (UBC) (see supplementary material for specific GC-MS methodology). All peaks found were integrated and tabulated as a percent value (Supplementary Fig. 1), and identified based on the Wiley/NIST (National Institute of Standards and Technology) Library matching. Sixteen individual compounds were identified, with the primary compounds (>2% of the total amount) being Menthone (55.61%) and its isomer (16.40%), Menthol (16.83%) and its isomer (3.42%), and Menthol acetate (2.741%). All other compounds and their percentages are listed in Table 1.

**Table 1:** Major compounds detected within the peppermint essential oil used within our bioassays, their retention time, and peak area percentages as determined by headspace volatile GC-MS analysis.

Compound	R_T_(min)	Peak area	Peak area %
Menthone	10.99	105423932	55.61
Menthol (isomer)	14.05	31916289	16.83
Menthone (isomer)	11.38	31095335	16.40
Menthol	13.12	6487428	3.42
Menthol acetate	12.61	5147243	2.71
Isopulegol	12.65	2280048	1.20
Pulegone	14.31	1742237	0.92
Piperitone	16.17	1354788	0.71
Menthan-8-ol	13.02	1155259	0.61
Limonene	6.84	656492	0.35
neo-Menthol	13.67	604178	0.32
Caryophyllene	13.4	546453	0.29
Menthan-4-ol	12.27	452935	0.24
Terpineol	15.26	308349	0.16
Cymene	7.8	230120	0.12
3-Methylcyclohexanone	8.69	191969	0.10

### Insect bioassays

We conducted four bioassays to assess the effects of fumigation with VOCs from peppermint essential oil on *D. suzukii* pupal survival and *P. vindemmiae* parasitism success. All bioassays with *D. suzukii* were conducted in an environmentally-controlled growth room at the University of British Columbia, Vancouver, Canada. Within the growth room, we maintained a 16:8 light:dark schedule with cool white lights. Temperatures within the growth room were 22.6 ± 0.4 °C and relative humidity was 46.5 ± 3.2%.

We prepared our peppermint essential oil fumigation treatments as described by Renkema *et al*.^19^. We prepared three peppermint essential oil stock solutions for use in all bioassays: (1) acetone (0 mg, control), (2) 15 g peppermint essential oil/L acetone, and (3) 30 g peppermint essential oil/L acetone. As determined through preliminary experimentation, we cut eRoma Microfiber Absorbing Pads into 1 × 1 cm squares and treated each pad with 150 μL of their respective treatment, via stock solution, before each experiment. All treated pads were placed in a fume hood for one hour to allow the acetone to evaporate before use in bioassays^19^. After the acetone evaporated, we were left with three fumigation treatments which we refer to by the mass of peppermint oil applied to the absorbing pad: 1) 0 mg (control), 2) 2.25 mg and 3) 4.5 mg.

#### Bioassay 1: Effects of peppermint oil VOC fumigation and soil moisture on D. suzukii emergence

As soil moisture and relative humidity can be important for *D. suzukii* survival, we sought to determine whether peppermint oil VOC fumigation would reduce *D. suzukii* emergence, and whether this depended on soil moisture. We placed two grams of dry potting soil in a 30 mL polystyrene cup (Solo), into which we added 2 mL of distilled water to half of the cups (moist soil treatment) and the others received no distilled water (dry soil treatment). We then covered the soil with a circle of Mosquito No-See-Um Ultra-Fine Netting (Skeeta) to prevent *D. suzukii* pupae and emerged adults from burying into the soil. Within each soil cup, we placed ten *D. suzukii* pupae. A treated pad with one of the three peppermint oil VOC fumigation treatments (0 mg (control), 2.25 mg, and 4.5 mg) was placed on the opposite side of the cup from the *D. suzukii* pupae (n = 20 for each treatment). Bioassay cups were covered with a perforated plastic lid, which allowed ventilation. We monitored and removed adult flies emerging within the cups every 24 hours until no more adult *D. suzukii* emerged (5–7 days).

#### Bioassay 2: Effects of peppermint oil VOC fumigation on parasitoid mortality and emergence

To determine whether peppermint oil VOC fumigation had a negative effect on *D. suzukii* emergence and *P. vindemmiae* parasitism, we conducted a bioassay with peppermint oil (0 mg (control) vs. 4.5 mg) and *P. vindemmiae* (male/female pair or none). We used these two concentrations based on preliminary analysis of Bioassay 1. We placed ten *D. suzukii* pupae along the side of a 120 mL clear plastic jar (Uline, Model S-9934) with ventilated lid, and placed treated pads on the opposite side of the jar from the *D. suzukii* pupae. Half of each peppermint VOC treatment received no wasps or one male/female *P. vindemmiae* pair (n = 15 for each treatment).

We recorded *P. vindemmiae* mortality every 24 hours and replaced dead wasps with new living wasps to maintain similar parasitism pressure. After 72 hours, we removed the remaining *P. vindemmiae*. *Drosophila suzukii* began emerging three days after the experiment start date with the majority of emergence happening on days four and five. We recorded *D. suzukii* emergence every 24 hours for one week until no more adult *D. suzukii* emerged. We continued to incubate the jars under the same laboratory conditions until *P. vindemmiae* emergence 23 days later. We counted and removed all emerging *P. vindemmiae* every 24 hours for six days, at which point no additional *P. vindemmiae* emerged.

#### Bioassay 3: Effects of peppermint oil VOC fumigation before parasitism

To identify whether VOCs produced by peppermint oil influenced *P. vindemmiae* host quality, we conducted a bioassay where we fumigated *D. suzukii* pupae with three different weights of peppermint oil (0 mg (control), 2.25 mg and 4.5 mg) prior to *P. vindemmiae* oviposition. We suspended a treated pad (0 mg (control), 2.25 mg or 4.5 mg peppermint oil) from the foam plug of a polystyrene Drosophila vial (VWR International, LLC.), into which ten *D. suzukii* pupae were placed. After 24 hours of peppermint oil VOC fumigation (or control), we removed the suspended treated pad and randomly placed either a single female *P. vindemmiae* or no wasp (control) into each peppermint oil VOC fumigation treatment (n = 15 per treatment).

We recorded *D. suzukii* emergence every 24 hours for one week until no more adult *D. suzukii* emerged. We continued to incubate the jars under the same laboratory conditions until *P. vindemmiae* emergence 23 days later. We counted and removed all emerging *P. vindemmiae* every 24 hours for six days, at which point no additional *P. vindemmiae* emerged.

#### Bioassay 4: Effects of peppermint oil VOC fumigation after parasitism

We conducted an additional bioassay to examine the effects of peppermint oil VOC fumigation (0 mg, 2.25 mg or 4.5 mg peppermint oil) on *D. suzukii* pupae after parasitoid oviposition. We placed ten *D. suzukii* pupae and one presumably mated *P. vindemmiae* female into a polystyrene Drosophila vial (VWR International, LLC.). After 24 hours for oviposition, we removed the *P. vindemmiae* female. We then suspended a treated pad (0 mg, 2.25 mg or 4.5 mg peppermint oil) from the foam plug of the bioassay vial (n = 15 for each fumigation treatment). As in previous experiments, we monitored *D. suzukii* total emergence and continued to incubate the jars under laboratory conditions until parasitoid wasp emergence. We counted and removed all emerging wasps every 24 hours until the end of wasp emergence.

### Statistical analysis

For *D. suzukii* and *P. vindemmiae* emergence, we used a generalized linear model (GLM) with a quasibinomial distribution and a log link function, after detecting overdispersion in preliminary models with binomial distribution. We then used F-tests to compare the effects among treatments^41^. For Bioassay 1, we found that *D. suzukii* emergence was zero for all replicates within one of the treatments (4.5 mg, dry soil). This confounded the model’s standard error estimates and our ability to conduct posthoc tests on interactive effects. To counter these issues, we treated peppermint oil weight as a continuous factor and soil moisture as a categorical factor for this analysis. For Bioassays 2-4, we analyzed the proportion of *D. suzukii* pupae that emerged as adults, dependent on peppermint oil weight, *P. vindemmiae* presence or absence, and their interaction as categorical factors. For *P. vindemmiae* treatments, we analyzed the proportion of *P. vindemmiae* emerged as adults from *D. suzukii* pupae, with peppermint oil weight as a categorical factor. To determine the effect of peppermint essential oil fumigation on *P. vindemmiae* death (Bioassay 2), we used a linear model with number of dead *P. vindemmiae* as the response variable and peppermint oil (as a categorical factor) as the explanatory variable. All analyses were conducted in R^42^.

## Results

### Bioassay 1: Effects of peppermint oil VOC fumigation and soil moisture on *D. suzukii* emergence

**Figure 1:**
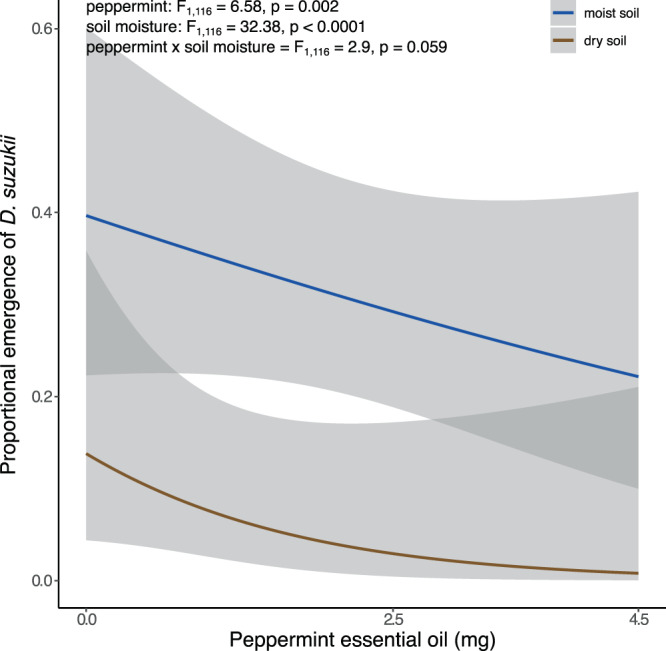
The proportion of adult *Drosophila suzukii* emerging from ten pupae experimentally fumigated with peppermint oil produced VOCs from three weights of peppermint essential oil (0 mg (control), 2.25 mg, and 4.5 mg). Curves depict continuous adjusted model means for proportional emergence with 95% confidence interval in moist (blue) or dry (brown) soil conditions.

The proportion of adult *D. suzukii* emergence decreased with exposure to peppermint oil VOCs through fumigation (peppermint: F_1,116_ = 6.58, p = 0.002; Fig. 1). The proportion of *D. suzukii* adults emerging was lower with dry soil compared to moist soil (soil moisture: F_1,116_ = 39.38, p < 0.0001; Fig. 1). In our control treatment, *D. suzukii* emergence decreased by 22% in dry compared to moist soil treatments. The effects of peppermint oil VOC fumigation was marginally significant in its interaction with the soil moisture treatment (peppermint x soil moisture: F_1,116_ = 2.89, p = 0.059, Fig. 1).

### Bioassay 2: Effects of peppermint oil produced VOCs on parasitoid mortality and emergence. 

**Figure 2:**
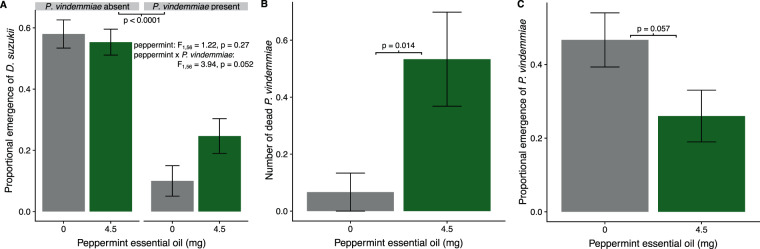
(**A**) The proportion of adult *Drosophila suzukii* emerging from ten pupae with or without the presence of the parasitoid wasp *Pachycrepoideus vindemmiae*, fumigated with VOCs from absorbance pads treated with two weights of peppermint essential (0 mg (control), and 4.5 mg); (**B**) the mortality of *P. vindemmiae* (number dead) dependent on peppermint oil VOC fumigation; (**C**) the proportion of *P. vindemmiae* emerging from ten possible pupal hosts fumigated with peppermint oil VOCs during parasitism. Means and standard error shown.

In the second experiment, fumigation with peppermint oil VOCs did not have an effect on *D. suzukii* emergence (peppermint: F_1,56_ = 1.22, p = 0.27, Fig. 2A). The presence of *P. vindemmiae* reduced *D. suzukii* emergence (*P. vindemmiae*: F_1,56_ = 45.59, p = <0.0001; Fig. 2A). The interaction between peppermint oil VOC fumigation and *P. vindemmiae* presence marginally affected *D. suzukii* emergence (peppermint x *P. vindemmiae*: F_1,56_ = 3.94, p = 0.052; Fig. 2A). When *P. vindemmiae* was present, we observed a difference in *D. suzukii* emergence of 15% between peppermint oil treatments, and a difference of 3% between peppermint oil treatments when *P. vindemmiae* was absent. *Pachycrepoideus vindemmiae* reduced *D. suzukii* emergence by 48% in 0 mg (control) and 30% in 4.5 mg peppermint oil VOC fumigation treatments. Fumigation with peppermint oil VOCs increased the number of dead *P. vindemmiae* during the oviposition phase of this trial (F_1,28_ = 6.86, p = 0.014; Fig. 2B) resulting in a marginal effect on subsequent *P. vindemmiae* emergence (F_1,28_ = 3.94, p = 0.057; Fig. 2C). In our 4.5 mg peppermint oil VOC fumigation treatment, subsequent *P. vindemmiae* emergence was reduced by 6%.

### Bioassay 3: Effects of peppermint oil VOC fumigation before parasitism

**Figure 3:**
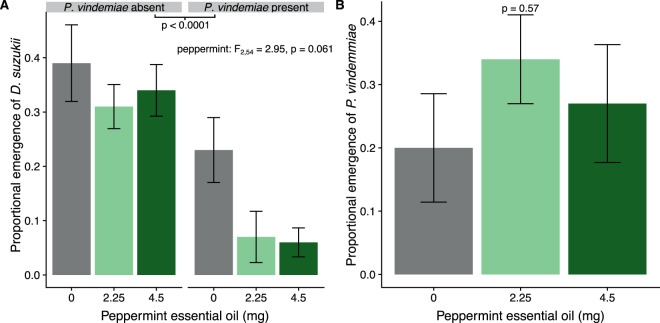
(**A**) The proportion of adult *Drosophila suzukii* emerging from ten pupae after 24 hours of pupal fumigation with VOCs from absorbance pads treated with three weights of peppermint essential oil (0 mg (control), and 4.5 mg) and subsequent exposure to the parasitoid *Pachycrepoideus vindemmiae* (presence or absence), with no significant interaction between peppermint oil and wasp presence; (**B**) The proportion of *P. vindemmiae* emerging from ten possible pupal hosts exposed to three peppermint essential oil fumigation treatments pre-oviposition. Means and standard errors shown.

In the third trial, pupal fumigation with peppermint oil VOCs reduced  *D. suzukii* emergence (peppermint: F_2,54_ = 2.95, p = 0.061; Fig. 3A), although the effect was only marginally significant. Independent of peppermint oil VOC fumigation, *P. vindemmiae* presence reduced *D. suzukii* emergence (*P. vindemmiae*: F_1,54_ = 25.7, p < 0.0001; Fig. 3A). The proportion of *D. suzukii* emergence ranged from 0.31 ± 0.032 and 0.39 ± 0.056 in peppermint oil VOC fumigation treatments without *P. vindemmiae*. Peppermint oil VOC fumigation and subsequent parasitism by *P. vindemmiae* did not have an interactive effect on *D. suzukii* (peppermint x *P. vindemmiae*: F_2,54_ = 2.04, p = 0.14; Fig. 3A). However, including *P. vindemmiae* reduced the proportion of *D. suzukii* emergence in 0 mg (control) to 0.23 ± 0.047, and 0.07 ± 0.037 and 0.06 ± 0.021 in 2.25 mg and 4.5 mg peppermint oil produced VOC treatments respectively. All adult *P. vindemmiae* used during parasitism survived, and fumigation with peppermint oil VOCs before parasitism had no effect on *P. vindemmiae* emergence (peppermint: F_2,27_ = 0.67, p = 0.52; Fig. 3B).

### Bioassay 4: Effects of peppermint oil VOC fumigation after parasitism

**Figure 4:**
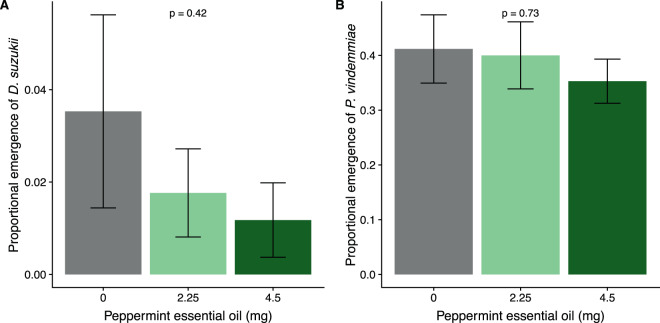
(**A**) The proportion of adult *Drosophila suzukii* emerging from ten pupae after 24 hours of parasitism by *Pachycrepoideus vindemmiae* and subsequent VOC fumigation with three weights of peppermint oil (0 mg (control), and 4.5 mg); (**B**) the proportion of adult *P. vindemmiae* emerging from ten pupae fumigated with three weights of peppermint oil after parasitoid exposure. Means and standard error shown, p-value shows main effect of peppermint oil.

In the fourth trial, we observed low *D. suzukii* emergence after parasitism with no effect of peppermint oil VOC fumigation (peppermint: F_2,48_ = 0.87, p = 0.42; Fig. 4A). Fumigation with peppermint oil VOCs after parasitism did not have an effect on *P. vindemmiae* emergence (peppermint: F_2,48_ = 0.32, p = 0.73; Fig. 4B).

## Discussion

Our study suggests that under certain conditions fumigation with peppermint oil VOCs may be effective at reducing *D. suzukii* during its pupal stage (Fig. 1), and moreover may be compatible with biological control (Figs. 3–4). We found fumigation of pupae with peppermint oil VOCs to be compatible with *P. vindemmiae* before and after oviposition; however, fumigation with peppermint oil VOCs was not compatible with adult *P. vindemmiae* actively parasitizing pupae (Fig. 2B,C).

While the specific chemical composition varies between essential oils, there is evidence that oils with higher concentrations of monoterpenoids, such as peppermint essential oil, are efficient adulticides and larvicides for insect pests^44^. However, while VOCs from peppermint oil has been shown to be repellent to adults^19,20^, its toxicity to Drosophila adults varies from no effect^19^, to increased mortality of *Drosophila auraria* (Peng) exposed to pennyroyal oil (*Mentha pulegium* L.)^45^. The effects of peppermint oil produced VOCs on *D. suzukii* were inconsistent across our bioassays, suggesting mixed efficacy of using peppermint oil VOCs to control pupal stages of *D. suzukii*. We found the strongest effect of peppermint oil fumigation within our first bioassay (Fig. 1), with marginal to no effect within the last three bioassays on *D. suzukii* emergence. Our results suggest that more intensive fumigation effects were seen in smaller chambers like the one used in our first bioassay. We suspect that these mixed results were dose dependent effects of peppermint oil VOCs on pupal survival that we were not able to fully capture with the current set of experiments. Similarly, although Renkema *et al*.^19^ found peppermint essential oil produced VOCs to be repellent for up to six days, a later study by Renkema *et al*.^20^ observed lesser peppermint oil VOC effects as they found that male repellence was lost at 24 hours and that peppermint VOCs were ineffective against females.

Our results suggest that soil moisture may be more important for *D. suzukii* pupal mortality than fumigation effects with peppermint oil VOCs (Fig. 1), and *D. suzukii* pupae on moist soil had 22% higher emergence rates compared to pupae on dry soils. As we did not measure humidity within the emergence cups directly, any difference seen between the moist soil and dry soil treatment could be due to either the change in soil moisture or the relative change in general humidity between the moist soil versus dry soil treatment. The effect of soil moisture we observed is consistent with others’ findings on the importance of microclimate and relative humidity is a key factor in *D. suzukii* success^23,24,43,46^. In the field, increased relative humidity increased *D. suzukii* fecundity and longevity while reduced humidity correlated with reduced trap captures^23^. Similarly, Diepenbrock and Burrack^24^ observed higher infestations of blackberry (*Rubus* L.) within the center canopy and around field edges with higher humidity levels. Under extreme dry conditions (0% moisture), larvae desiccate or pupate on the soil surface and pupate at a shallow soil depth (1–6 mm) when moist conditions are present^46^.

Irrigation regimes are regionally tailored for specific crops. In berries, irrigation methods are often implemented for quality (i.e. firmness^47^) or yield^48,49^, with consideration for disease incidence such as root rot^50,51^. In regard to *D. suzukii*, higher pupal emergence is associated with overhead sprinklers compared to drip irrigation as pupae located on the soil surface are less likely to desiccate with overhead irrigation systems^43^. In our study, there was a marginal interactive effect between soil moisture and peppermint oil VOC fumigation. Exploiting the potential general interaction between soil moisture and other control efforts may reduce *D. suzukii* in the field, as we found that fumigation effects of peppermint oil were especially effective in dry conditions. During suboptimal conditions, like drought, fumigation with peppermint essential oil may be a better tactic than when more optimal conditions are present. When conditions are optimal for soil pupation, such as when growers utilize overhead sprinkler irrigation, peppermint oil fumigation could still potentially reduce *D. suzukii* levels, but more work is needed to determine an effective application rate beyond what we tested and determine any potential non-target effects.

We did not test how moisture levels influenced *P. vindemmiae*’s response to the presence of peppermint oil VOCs. Moreover, the *P. vindemmiae* in our three bioassays did not have a water or sugar source during their oviposition time, potentially influencing their behaviour and effect on *D. suzukii* mortality. For example, Da Silva *et al*.^52^ found that water-deprived *P. vindemmiae* increased their host-feeding behaviour, resulting in increased *D. suzukii* mortality. Furthermore, this host-feeding behaviour lead to increased parasitism and had no effect on *P. vindemmiae* offspring mortality^52^. In a different study, Da Silva *et al*.^53^ found that a sugar food source (i.e. honey) for young *P. vindemmiae* females, like those we used in our bioassays, did not affect *D. suzukii* host mortality.

Integrated pest management plans which use VOCs may be more compatible with biological control options than conventional insecticides^15,54^, motivating our study to observe the effects of peppermint oil VOC fumigation on *P. vindemmiae* performance. Our results show that for peppermint oil produced VOC, the timing of application is important for its compatibility with biological control. We found that direct fumigation with peppermint oil VOCs to be toxic to adult *P. vindemmiae*, while indirect effects of peppermint oil VOCs on *P. vindemmiae* were minimal (i.e. through potential changes in pupal host quality from peppermint oil VOCs exposure before and after parasitism). This may be because *P. vindemmiae* is able to complete development even within dead pupae, as we have found that *P. vindemmiae* successfully emerge from previously frozen *D. suzukii* pupal hosts (Reut, personal observation). Additionally, these results suggest that any persistence of peppermint oil VOCs on pupal hosts is short-lived and unlikely to directly expose *P. vindemmiae* to residual fumigation. Overall, high mortality and decreased parasitism rates occur in *P. vindemmiae* when directly exposed to insecticides, including spinosyns, abamectin, neonicotinoids, organophosphates and pyrethroids^39^. Furthermore, spinosad-treated pupae result in increased mortality of adult *P. vindemmiae* females^55^. We found fumigation with peppermint oil VOCs to be toxic to adult *P. vindemmiae* indicating that peppermint oil VOC fumigation should not occur when adult *P. vindemmiae* are active within the field.

While adult *P. vindemmiae* were negatively affected by peppermint oil VOC fumigation, we found developing *P. vindemmiae* receive some protection during peppermint oil VOC fumigation while within *D. suzukii* pupal cases. Other immature parasitoids benefit from insecticide protection while maturing within their hosts as has been shown with the housefly, *Musca domestica* L.^56^, aphids and mealybug mummies^57–59^. Insecticide protection during development within *D. suzukii* hosts has also been shown in *P. vindemmiae* but that degree of protection is dependent on timing of development^55^. Further understanding of the timing of peppermint oil VOC fumigation and associated trade-offs are needed in order to further develop *D. suzukii* management plans.

More research is needed to determine how peppermint oil VOC fumigation reduces *D. suzukii* emergence from its pupal stages in field applications. More localized applications of peppermint oil VOCs at the soil surface may be accomplished by using laminate flake technology. For example, laminate polymer flakes treated with peppermint oil have been shown to decrease *D. suzukii* oviposition in strawberries with recommendations for frequent applications (at least every four days)^20^. However, the effects of laminate flake technology treated with essential oils on *D. suzukii* pupae in field applications have yet to be determined. This technology could potentially provide an additional management strategy through more localized applications of peppermint oil VOCs. Furthermore, it could be especially useful for farmers using overhead sprinkler irrigation, as this would exploit the potential interactive effect of soil moisture with peppermint oil produced VOCs.

## Conclusion

Our results provide further support that microclimatic effects (e. g. humidity) are important for *D. suzukii* control and reducing humidity in the field will help suppress *D. suzukii* populations and potentially amplify effects of other control efforts. Using peppermint essential oil as a source of VOCs may reduce *D. suzukii* emergence from the pupal stage, and these techniques could potentially be used alongside a biological control program with *P. vindemmiae* or other natural enemies.

## Supplementary information

Essential Oil GC-MS.

## Data Availability

The datasets generated during the current study are available from the corresponding author upon reasonable request.
