# Supplementary material for: Peppermint essential oil inhibits Drosophila suzukii emergence but reduces Pachycrepoideus vindemmiae parasitism rates
Source: Sci Rep. 2020 Jun 4;10:9090. doi: 10.1038/s41598-020-65189-5 (PMC7272437; doi:10.1038/s41598-020-65189-5)
Supplement: Supplementary file 1 — Essential Oil GC-MS. [file 41598_2020_65189_MOESM1_ESM.pdf]

Peppermint essential oil inhibits *Drosophila suzukii* emergence but reduces *Pachycrepoideus vindemmiae* parasitism rates

Chelsea Megan Gowton,[1*] Michał Reut[1,2] and Juli Carrillo[1]

1 Faculty of Land and Food Systems, Centre for Sustainable Food Systems, Biodiversity Research Centre, The University of British Columbia, Unceded xʷməθkʷəy̓əm (Musqueam) Territory, Vancouver, V6T 1Z4 British Columbia, Canada.

2 Department of Applied Entomology, Faculty of Horticulture and Landscape Architecture, Warsaw University of Life Sciences – SGGW, Nowoursynowska 159, 02-776 Warsaw, Poland

*Corresponding author: cgowton@mail.ubc.ca

ORCID:

Chelsea Megan Gowton: 0000-0003-2443-0809

Michał Reut: 0000-0002-9259-7830

Supplementary Materials

Essential Oil GC-MS methodology

We prepared our headspace sample by placing one µL of undiluted peppermint essential oil in a 10.0 ml headspace vial and incubated the sample for 10 min at 30° C. A volume of 500 ul of headspace sample was injected into an Agilent 6890N/5973 Gas Chromatograph Mass Spectrometer system.

The GC initial oven program temperature was 40° C, held for 3 minutes, then ramped to 100C at 10° C/minute, then ramped to 130°C at 3C/min, and finally ramped to a final temperature of 250C at 30C/min and hold for 12 minutes. The total run time was 35 min. The injection was done in split mode with a ratio of 5:1. The column used was an Agilent DB-Wax column (P/N 122-7062) with a dimension of 60 m x 0.25 mm id and 0.25 µm film thickness. The carrier gas was Helium and set at a flow rate of 1.9 mL/min. The mass spectrometry analysis was run in electron ionization mode at 70eV with a mass scan range of 35-500. Mass spectra were match against Wiley 09/NIST 08 libraries.

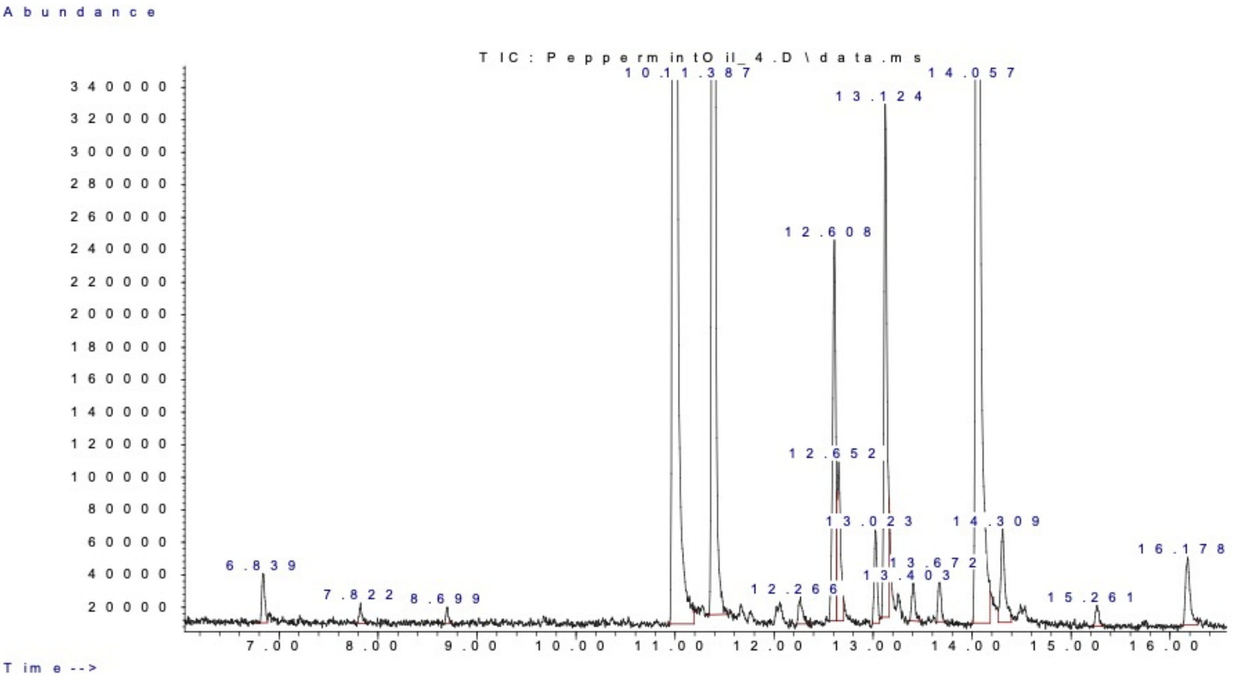

Supplementary Figure 1: The representative chromatogram of headspace volatiles from a commercially available peppermint essential oil. Compound identification numbers correspond to retention times listed in Table 1.
